# Biosafety and biosecurity for *Burkholderia pseudomallei* and *Burkholderia mallei*: evidence, gaps, and sustainable practice in endemic, low-resource settings

**DOI:** 10.1128/cmr.00369-25

**Published:** 2026-03-11

**Authors:** Stuart D. Blacksell, Khanh Kim Le, Sandhya Dhawan, Vanaporn Wuthiekanun, Direk Limmathurotsukul, Susanna J. Dunachie, Robert Norton, Ella Meumann, Bart J. Currie, Nicholas P. J. Day, David A. B. Dance

**Affiliations:** 1 Mahidol Oxford Tropical Medicine Research Unit, Faculty of Tropical Medicine, Mahidol University 26685, Bangkok, Thailand; 2 Centre for Tropical Medicine and Global Health, Nuffield Department of Medicine, University of Oxford 6396, Oxford, United Kingdom; 3 Pathology Queensland, Townsville University Hospital 830031 , Townsville, Queensland, Australia; 4 Global and Tropical Health Division, Menzies School of Health Research, Charles Darwin University 10095, Darwin, Northern Territory, Australia; 5 Department of Infectious Diseases, Royal Darwin Hospital 71507, Darwin, Northern Territory, Australia; 6 Microbiology Department, Territory Pathology, Darwin, Northern Territory, Australia; 7 Faculty of Infectious and Tropical Diseases, London School of Hygiene and Tropical Medicine 4906, London, United Kingdom; University of Pittsburgh School of Medicine, Pittsburgh, Pennsylvania, USA; Monash University, Melbourne, Australia; Public Health England, Salisbury, United Kingdom

**Keywords:** *Burkholderia pseudomallei*, *Burkholderia mallei*, biosafety evidence

## Abstract

*Burkholderia pseudomallei* and *Burkholderia mallei* present significant biosafety challenges due to their high pathogenicity, environmental resilience, intrinsic antimicrobial resistance, and potential use as bioterrorism agents. This review examines key aspects of laboratory management and infection control for these organisms, focusing on inconsistencies in biosafety protocols and risk classifications across regions. We synthesize current evidence on biocontainment requirements, disinfection strategies, and personal protective equipment (PPE), with particular emphasis on sustainable practices for laboratories in low-resource settings. Although laboratory-acquired infections are rare, their potential severity underscores the importance of stringent safety measures. Critical gaps remain in our understanding of infectious dose, the effectiveness of post-exposure prophylaxis (PEP), and the development of risk assessment frameworks. We advocate for harmonized global biosafety standards and targeted research on transmission dynamics and inactivation protocols. These priorities are essential to enhance laboratory safety, especially in endemic areas, and to inform coherent international policy on *B. pseudomallei and B. mallei* containment and management.

## INTRODUCTION

*Burkholderia pseudomallei* and *Burkholderia mallei* are important pathogens due to their high virulence, environmental resilience, intrinsic antimicrobial resistance, and potential use as biothreat agents. These organisms pose biosafety and biosecurity challenges, particularly in laboratory settings where handling procedures must be implemented to mitigate the risk of accidental exposure or environmental contamination. Managing these risks is especially critical in low-resource tropical settings, where *B. pseudomallei and B. mallei* are frequently or occasionally isolated, but containment measures are limited.

Internationally, the World Health Organization (WHO) (1, 2) and the World Organisation for Animal Health (WOAH) (3) aim to promote an evidence-based approach to biological risk management through their biosafety and biosecurity reference publications, focusing on sustainable laboratory practices and engineering controls. To this end, the WOAH Biosafety Research Roadmap project (4) has spearheaded efforts to identify research gaps, strengthen sustainable biosafety practices, support laboratory biological risk management applications, enhance laboratory sustainability, and provide an evidence-based approach to biosafety measures, including engineering controls, particularly in low-resource settings.

This review provides a detailed examination of the characteristics of *B. pseudomallei* and *B. mallei*, describing biosafety and biocontainment practices while underscoring knowledge gaps and research needs to support safer laboratory practices and global health security with a focus on low-resource settings.

## GENERAL CHARACTERISTICS

The *Burkholderia* genus includes a group of Gram-negative, obligate aerobic bacteria, most of which are motile. Among these, *B. pseudomallei* is distinguished by its role as the causative agent of melioidosis, a disease affecting humans and numerous animal species. This species thrives as a sapronotic soil bacterium, with its viability influenced by various physicochemical properties of the surrounding soil (5). In contrast, *B. mallei*, a non-motile bacterium which is a clonal derivative from an ancestral *B. pseudomallei*, primarily causes glanders in equines and can also infect humans (6–8). Both *B. pseudomallei* and *B. mallei* are regarded as potential bioterrorism agents (9).

### Epidemiology

*B. pseudomallei* has a global distribution, predominantly in tropical and subtropical regions, including South and Southeast Asia, northern Australia, Africa, and parts of Central and South America (10–12). In contrast, *B. mallei* has been largely eradicated worldwide (13). However, it remains enzootic and is reemergent in some regions of Asia, the Middle East, Africa, Central America, and South America (8, 10, 14).

### Mode of transmission

In endemic regions, *B. pseudomallei* is widely distributed in soil and surface water, increasing the risk of infection for individuals with recreational and occupational exposure, such as farmers and herders (15). Transmission to humans and animals can occur through skin wounds or lesions, inhalation of aerosols (often generated during severe weather), or ingestion of contaminated water (16). Zoonotic transmission of *B. pseudomallei* is extremely uncommon. Conversely, *B. mallei* can be transmitted to individuals in close contact with infected animals (e.g., grooms, veterinary surgeons) via open skin wounds, inhalation of aerosols, or ingestion of contaminated food (7, 13, 17). As with *B. pseudomallei*, human-to-human transmission of *B. mallei* is extremely rare (13, 17, 18). Compared to other members of the *Burkholderia* genus, the exceptionally virulent *B. mallei* is a zoonotic pathogen that is not capable of long-term environmental persistence in soil or water. Instead, it relies on animal hosts, including equines, felines, camelids, ursids, and canids, which serve as reservoirs for the bacterium (6, 13).

### Infectious dose

The infectious doses of *B. pseudomallei* and *B. mallei* remain poorly defined, with most data derived from animal studies that demonstrate considerable variability depending on both the bacterial strain and the host species. In mice, some studies report that as few as 100 colony-forming units (CFU) can establish chronic infection (19). Non-human primate studies show that *B. pseudomallei* can cause acute, severe melioidosis following very low-dose exposures, particularly via the aerosol route (20, 21). These models demonstrate high susceptibility and strain-dependent virulence, reflecting the organism’s ability to initiate infection from minimal exposure (22).

### Diagnosis and treatment

Bacterial culture remains the gold standard for diagnosing infections caused by *B. pseudomallei* and *B. mallei* (23). Current recommendations for the treatment of melioidosis are informed by randomized controlled trials conducted in Thailand and cohort studies in northern Australia, which have significantly reduced mortality rates. The treatment protocol consists of two phases: an intensive phase, typically lasting 10–14 days, involving intravenous antibiotics like ceftazidime or meropenem to control the acute infection, followed by an eradication phase lasting 3–6 months with oral antibiotics, primarily trimethoprim-sulfamethoxazole, to prevent relapse. When resources permit, the duration of the intensive phase may be extended to further reduce the risk of relapse, which aligns with the revised Darwin guidelines (24). Integrating these guidelines with optimal sepsis management has shown remarkable success; for instance, Darwin has seen a decline in melioidosis case fatality ratio to just 6% over the past 5 years (25); however, the rate remains high in many endemic countries. There is no vaccine available against *Burkholderia* spp., although several research groups are investigating potential options (26–31).

## BIOSAFETY CHARACTERISTICS

### Risk group classification

The classification of *B. pseudomallei* and *B. mallei* into risk groups (RG) varies by jurisdiction. Generally, both are categorized as RG3 pathogens for humans and animals in regions such as the UK (32), the USA (33), Switzerland (34), and the European Union (35). In the UK, *B. mallei* is also regulated under the Specified Animal Pathogens Order (32). In Australia, however, *B. pseudomallei* is classified as RG2 and requires Biosafety Level (BSL) 2 containment (36), and *B. mallei* is designated as RG3 (36)*.*

### Biothreat potential and risks of *B. pseudomallei* and *B. mallei*

*B. pseudomallei* and *B. mallei* have intrinsic characteristics that make them potential bioterrorism agents. In 2002, the US CDC classified both as Category B biothreat agents based on their potential for dissemination, moderate morbidity, and relatively low mortality (9, 37). Additionally, *B. mallei* and *B. pseudomallei* are designated as Tier 1 Select Agents under the Code of Federal Regulations (42 CFR Part 73) (38), which imposes stringent guidelines on the possession, storage, use, and transfer of these pathogens (8, 39). In Singapore, both are listed as First Schedule Part II Pathogens (40). In contrast, Australian regulatory frameworks do not classify *B. pseudomallei* or *B. mallei* as Security Sensitive Biological Agents (SSBA) (41), most likely reflecting practical considerations associated with the endemicity of *B. pseudomallei* in northern Australia and its common occurrence in routine diagnostic microbiology.

### Laboratory exposures and infections

#### Laboratory-acquired infections

While surveillance data show that exposures are relatively common, Table 2 illustrates that these exposures progress to laboratory-acquired infections (LAIs) only rarely, usually in the context of significant laboratory safety lapses or predisposing host factors. Only two published cases of laboratory-acquired *B. pseudomallei* infections have been reported, both following significant lapses in laboratory practices. In 1966, a researcher developed pneumonia after cleaning a *B. pseudomallei* spill with her bare hands despite an ulcerative lesion on her hand, likely contracting the infection via inoculation or inhalation (42). In 1980, a laboratory technician, initially thought to be unexposed, was infected after sonication of a misidentified *B. pseudomallei* isolate in an open flask, with inhalation suspected as the route of infection (43). In addition, the authors are aware of seven further unpublished cases among staff in diagnostic and research laboratories in Asia. Six of the staff concerned had been involved in the lyophilization of *B. pseudomallei* or glassware cleaning. In a seventh worker who primarily handled mycobacterial culture samples, localized melioidosis might have resulted from laboratory exposure to respiratory tract samples containing *B. pseudomallei*. However, in all these cases, endemic exposure cannot be ruled out (V. Balaji, E. Corea, and B. Piyasiri, personal communications). Data on strain identity in documented laboratory-acquired infections are limited, precluding robust assessment of strain-dependent virulence in occupational exposure. Where reported, infections have involved diverse strains, suggesting that host factors and exposure route may play a more significant role than strain alone.

Historically, nine fatal laboratory-acquired *B. mallei* infections were documented between 1905 and 1925, primarily resulting from centrifuge accidents, broken culture tubes, and exposure to infected laboratory animals (44). In 1944–1945, six cases emerged in a single laboratory linked to accidental culture spills and aerosol exposures (45). With glanders in horses becoming rare, exposure opportunities to *B. mallei* declined; however, in 2000, a research microbiologist at the United States Army Medical Research Institute of Infectious Diseases (USAMRIID) contracted a *B. mallei* infection, possibly due to inconsistent glove use and an increased susceptibility resulting from diabetes (46). These cases suggest that *B. mallei* may pose a greater laboratory hazard than *B. pseudomallei*, though historical reports reflect an era with fewer biosafety precautions.

#### Laboratory exposures

Analysis of U.S. surveillance data reveals that exposure to *B. pseudomallei* is relatively common, but it rarely results in LAIs (47) (Table 1). Data from the Melioidosis Intake Database (MID, 2008–2014) show that most incidents were low risk, such as accidental plate openings (33%), sniffing colonies (9%), or minor spills, with 34% involving higher-risk aerosol-generating activities outside biological safety cabinets (48–50). The Morbidity and Mortality Weekly Report (2008–2013) identified 261 laboratory staff members at risk, of whom 16% had high-risk exposures, 50% had low-risk exposures, and 34% had undetermined exposures; none developed melioidosis. More recently, Clay et al. (2017–2019) documented 51 release events involving 275 individuals, with 84% occurring outside primary containment and mainly attributed to human error, including spills, needlesticks, personal protective equipment (PPE) failures, and equipment malfunctions; again, no infections were reported (51). Finally, Richardson et al. (2008–2024) described 160 exposure events affecting 855 personnel, of whom 12% developed positive IHA titers, and five showed fourfold rises. Still, none developed disease, even though most exposures (69%) and all high-risk incidents occurred outside biological safety cabinets (52). Taken together, these findings reinforce the conclusion that while exposures are not infrequent, particularly outside containment, confirmed laboratory-acquired melioidosis remains rare, underscoring the importance of strict biosafety practices and regular staff awareness training (51, 53).

**TABLE 1:** Risk assessment of laboratory incidents involving *B. pseudomallei* using the consensus risk classification adapted from Peacock et al. (54) and the observed exposures not resulting in clinical disease from U.S. surveillance data between 2008 and 2019 (47, 51, 52)^*e*^^,^

Risk category	Activity	Details/definition	Frequency observed			
			United States, 2008–2014, Richardson et al.^*a*^	United States, 2008–2013, Benoit et al.^*b*^	United States, 2017–2019, Clay et al.^*c*^	Australia, Gassiep et al., 2021^*d*^
Low risk	Accidental opening of agar plate	Unintentional opening of an agar plate containing *B. pseudomallei* outside a BSC	18 (33%)	Included under 130 low-risk exposures	–^*f*^	–
	Unintentional sniffing of agar plate	Sniffing of colonies without direct contact	5 (9%)	As above	–	–
	Splash exposure to protected skin	Splash to gloved hand or protected body; no aerosol	2 (4%)	As above	–	–
	Small-scale spill	<1 mL spill inside a functioning BSC	1 (2%)	As above	–	–
	Intact skin contamination	Contact of culture with intact skin	–	As above	–	–
	Unspecified/unknown	Reported as low-risk but activity is not specified	9 (16%)	As above	–	–
	General low-risk exposures	As classified in surveillance review	–	130/261 (50%)	–	–
	Routine manipulations outside BSC (oxidase, catalase, McFarland suspension, plate opening)	Handling *B. pseudomallei* isolates outside a biosafety cabinet in endemic-region diagnostic labs	–	–	–	1,267/1,419 handling events (89%) outside BSC; 0 infections; 0 seroconversions
High risk	Splash to mucous membranes	Splash to mouth/eyes (outside of BSC)	1 (2%)	Included under 43 high-risk exposures	–	–
	Aerosol generation outside BSC	Sonication, centrifuge incident, work outside containment	19 (34%)^*e*^	43/261 (16%) high-risk exposures	43/51 releases (84%) outside containment	–
	Needlestick or penetrating injury	Injury with contaminated sharp instrument	–	Included under 43 high-risk exposures	Two events	–
	Animal bite or scratch	Bite/scratch from infected experimental animal	–	As above	–	–
	Predisposing conditions without PPE	Handling with underlying health risk factors (e.g., diabetes, liver/kidney disease)	–	–	–	–
	PPE failure	Failure of PPE integrity or doffing	–	–	Two events	–
	Spill (larger-scale)	Release of culture outside BSC (beyond small volume)	–	–	Two events	–
	Equipment/mechanical failure	Failures of containment equipment or effluent systems	–	–	One event	–
	Failure to follow lab practices	Deviations from SOPs, communication failure	–	–	One event	–
Unknown	Risk not determined	Exposure reported but risk level not classified	–	88/261 (34%)	–	–

^*a*^ Frequency data (percentages and counts)—U.S. Melioidosis Intake Database (MID, 2008–2014) and IHA seroconversion logs (2009–2024) reported by Richardson et al. (52).

^*b*^ Benoit et al. (47).

^*c*^ Clay et al. (51).

^*d*^ Gassiep et al. (55).

^*e*^ Five laboratory personnel had a ≥4-fold rise in IHA titers (≥1:40); none developed melioidosis (52).

^*f*^ –, no data.

Unrecognized exposures occur when laboratory staff handle *B. pseudomallei* without realizing its identity at the time, resulting in reduced protective measures. In endemic areas, such exposures are common but almost never result in infection among laboratory staff (56). Many unrecognized exposures occurred due to misidentification of *B. pseudomallei* as other closely related organisms, such as *Burkholderia thailandensis* (57) or *Burkholderia cepacia* complex (50, 58), leading to reduced protective measures, and, in some cases, post-exposure prophylaxis was initiated only after *B. pseudomallei* was confirmed. A recent study highlighted the relative safety of handling *B. pseudomallei* outside biological safety cabinets in Australia. Among 30 laboratory scientists who handled *B. pseudomallei* on 1,267 occasions outside such containment, no infections occurred, and all participants remained seronegative. Furthermore, air sampling during 78 handling events, including plate opening, oxidase testing, and McFarland suspension preparation, showed no aerosolization of the organism (55). Periodic serological monitoring has been employed in some laboratory surveillance programs; however, the interpretation of isolated seroconversion remains uncertain. Serological results should therefore be interpreted cautiously and in conjunction with documented exposure history, rather than used as a stand-alone indicator of infection.

### Risk mitigation strategies

#### Handling *Burkholderia* spp. in research and diagnostic settings

Risk mitigation strategies for *B. pseudomallei* and *B. mallei* should be framed within the hierarchy of controls, which prioritizes elimination and substitution where feasible, followed by engineering controls, administrative measures, and, finally, PPE. In laboratory practice, engineering controls such as biological safety cabinets and workflow design provide more reliable protection than reliance on PPE alone, particularly in resource-limited settings where compliance and supply may be inconsistent. Centrifugation represents a recognized high-risk laboratory activity due to the potential for aerosol generation, particularly in the event of tube breakage or rotor failure. Sealed rotors or safety cups are strongly recommended, and loading and unloading should be performed within a biological safety cabinet. Rotors should only be opened after adequate settling time to minimize aerosol exposure.

Because laboratory-acquired melioidosis and glanders remain rare but potentially severe, biosafety efforts should focus on preventing exposures that could lead to infection. Laboratories must comply with the biosafety regulations and guidance established by the competent authorities in the countries where they operate. These frameworks define the appropriate containment levels, facility standards, and personal protective measures required for safely handling *B. pseudomallei* and *B. mallei*. For example, in the United States, the Centers for Disease Control and Prevention’s *Biosafety in Microbiology and Biomedical Laboratories, 6th edition (BMBL6*), recommends Biosafety Level 3 (BSL-3) and Animal Biosafety Level 3 containment for activities such as culture handling, animal necropsy, and experimental infection studies (33). Comparable national regulations elsewhere should be followed to ensure safe laboratory practices, including the use of appropriate containment equipment, facilities, and PPE, such as respirators, gloves, gowns, and eye protection.

#### Personnel restrictions for working with *B. mallei* and *B. pseudomallei*

Comprehensive consensus guidelines have been established regarding personnel restrictions for working with *B. mallei* and *B. pseudomallei* (54) (see Table 2). Staff with predisposing conditions, such as diabetes mellitus, chronic liver or kidney disease, hazardous alcohol use, long-term steroid use, hematologic malignancies, neutropenia or neutrophil dysfunction, chronic lung diseases (including cystic fibrosis), thalassemia, or any form of immunosuppression, should be considered for restriction or counseling before working with these pathogens (54). Additionally, laboratory access and data should be restricted to authorized personnel only. Personnel restrictions should be implemented through proportional, individualized occupational health risk assessments that consider comorbidities, training, experience, and the specific activities performed, rather than applying blanket exclusions, and should align with local occupational health and safety frameworks.

**TABLE 2:** Summary of published laboratory-acquired infections with *B. pseudomallei* and *B. mallei*: causes, risk factors, and outcomes

Year	Agent	Case(s)	Cause/exposure activity	Risk factors/notes	Outcome	Reference
1966	*B. pseudomallei*	One case, Toronto	Centrifuge leak in continuous-flow centrifuge; aerosolized culture sprayed into lab	Acute exposure to concentrated aerosols	Hospitalized with pneumonia; survived	(42)
1980	*B. pseudomallei*	One case	Aerosols generated during antimicrobial susceptibility testing (open flask sonication)	Inhalation of infectious aerosols	Hospitalized with pneumonia-like illness; survived	(43)
1905–1925	*B. mallei*	Nine fatal cases	Centrifuge accidents; broken culture tubes; inoculation accidents (guinea pig autopsy)	Early laboratories with no biosafety precautions; frequent injuries	Nine deaths	(44)
1944–1945	*B. mallei*	≥6 documented cases	Flask breakage (aerosol), animal inoculation, plating, serological work, routine culture transfers	Multiple exposures; no modern PPE; repeated handling of virulent cultures	Several hospitalized; some fatal	(45)
2000	*B. mallei*	One case, USAMRIID	Routine microbiology research; inconsistent glove use; inhalation or skin contact suspected	Patient had type 1 diabetes mellitus (predisposing factor)	Survived after severe illness	(46)

Given the biothreat potential of these pathogens, it is essential to implement robust physical and cybersecurity measures as part of a comprehensive biosecurity framework.

### Management of laboratory incidents and accidents

As with all laboratory biosafety activities, it is essential to thoroughly assess the hazards and risks associated with working with *B. pseudomallei* or *B. mallei* and implement locally feasible and effective mitigation measures. Detailed plans and procedures should also be established to manage accidental exposure. Wherever possible, planning should involve relevant occupational health services to ensure they are aware of the necessary actions in the event of an exposure incident. Expert consensus recommendations by Peacock et al. provide valuable guidance in this area (54). In the immediate aftermath of a laboratory incident or accident involving skin exposure to or inoculation of *B. mallei* or *B. pseudomallei*, the contaminated site should be washed promptly with water and an appropriate sanitizer such as 2%–4% Chlorhexidine (59) or 60%–70% ethanol or isopropanol (60). The incident should be reported to a supervisor and a safety officer, and the type of material involved should be documented. A trained spill-response team should then apply effective disinfectants with recommended concentrations and contact times (Table 3). Risk assessments, adapted from Peacock et al. (54), classify laboratory incidents involving *B. pseudomallei* as either high or low risk (see Table 1). High-risk exposures include inhalation, punctures, or contact with aerosols in the eyes. However, all exposure incidents should be approached with appropriate caution. When in doubt about the need for further action, the individual should be referred to the local occupational health service, if one is available.

**TABLE 3:** Summary of effective chemical, gas, and physical inactivation concentrations and contact times for *B. pseudomallei* and *B. mallei*

Methodology	Treatment	Concentration /condition	Exposure time	Application/findings	Organism(s) tested	Reference
Chemical	Chlorine	1 mg/L	Immediate	~99.99% reduction in water	*B. pseudomallei*	(59)
	Chlorine	0.5 mg/L	10 min	~2 log₁₀ reduction in potable water	*B. pseudomallei*	(61)
	Bleach (pH-adjusted)	~0.5% NaOCl, pH 6.8	15–30 min	>6 log₁₀ reduction on non-porous; variable on porous	*B. pseudomallei* (1026b)	(60)
	Chlorine dioxide	0.25 mg/L	10 min	≥3 log₁₀ reduction; potable water disinfection	*B. pseudomallei*	(62)
	Ethanol	70%	Immediate	>6 log₁₀ reduction on glass and aluminum; variable on porous	*B. pseudomallei* (1026b)	(60)
	Umonium^38^	≥0.5%	15 min	Effective; residual up to 14 days	*B. pseudomallei*	(63)
	Perasafe	0.2%	Immediate	Effective for equipment	*B. pseudomallei*	(64)
	PineSol	Undiluted	15–30 min	>6–7 log₁₀ reduction non-porous; 4–5 log₁₀ on wood	*B. pseudomallei* (1026b)	(60)
	Quaternary ammonium	Undiluted	15–30 min	>6 log₁₀ on glass and aluminum; up to 5 log₁₀ on wood	*B. pseudomallei* (1026b)	(60)
	Virkon	1%	30 min	Effective with extended contact	*B. pseudomallei*	(63, 64)
	Paraformaldehyde (cells)	4% PFA	5–15 min	Complete inactivation of infected cells; 4% for 15 min recommended SOP	*B. pseudomallei* (1026b)	(65)
	Formaldehyde (tissues)	4% (10% NBF)	~14–21 days	Complete inactivation of infected spleen, liver, lung, skin	*B. pseudomallei* (1026b)	(65)
	Electron microscopy fixative (tissues)	4% PFA + 1% glutaraldehyde	~7 days	Complete inactivation of tissues for electron microscopy	*B. pseudomallei* (1026b)	(65)
Gas/vapor	Hydrogen peroxide vapor/peracetic acid vapor	Standard conc.	Several hours	Used for fumigating labs; effective against vegetative Gram-negatives	*B. pseudomallei*	(1, 33)
	Formaldehyde (fumigation, historical)	Variable	Hours	Used historically for laboratory decontamination; now replaced by safer methods	General pathogens including *Burkholderia*	(1)
Physical	Heat (sub-autoclave)	74°C–80°C	10–60 min	Killed at 74°C (10 min) or 80°C (1 h)	*B. pseudomallei*	(66)
	Autoclaving	121°C, 103 kPa	15 min	Standard sterilization; complete inactivation	*B. pseudomallei, B. mallei*	(13, 33, 66)
	UV (artificial)	1–5.5 mJ/cm²	Immediate	1–4 log₁₀ reduction depending on strain	*B. mallei* (M-9, M-13)	(67)
	UV (monochloramine + UV)	10⁶ CFU/mL	Immediate	99.9% kill by monochloramine; 99% by UV	*B. pseudomallei*	(64)
	UV (sunlight)	Solar noon exposure	60–180 min	10⁵ CFU killed in 60 min; 10⁶ CFU in 180 min	*B. pseudomallei*	(68)
	Gamma irradiation	High-dose (kGy)	Variable	Sterilization of research material and equipment	*B. pseudomallei*	(69)

### Post-exposure prophylaxis

Consideration should be given to the use of post-exposure prophylaxis (PEP) for personnel involved in high- or low-risk exposure incidents, after thoroughly explaining the associated risks and benefits. However, there is no conclusive evidence that PEP will reduce the risk of infection, and the agent usually recommended, trimethoprim-sulfamethoxazole, carries a risk of side effects which may sometimes be severe (54, 70). Generally, all workers involved in a high-risk incident should be offered PEP immediately. For individuals involved in a low-risk incident, the decision to offer PEP should be based on the presence of known risk factors for naturally occurring melioidosis. Persons with such risk factors should be offered PEP, whereas those with no known risk factors should be managed with postexposure monitoring alone. PEP should be prescribed only by a physician (e.g., an occupational health service) who can provide ongoing monitoring for any adverse drug effects (54). The decision to administer PEP should carefully weigh the risk of infection, considering both the type of exposure and individual susceptibility, against potential side effects, which can be significant enough to require cessation or change of treatment (71, 72). Specific dosage and frequency guidelines for *B. pseudomallei* PEP are detailed by Peacock et al. (54). If PEP is deemed appropriate after discussing these factors with the exposed individuals, prompt administration is recommended, typically using trimethoprim/sulfamethoxazole, or doxycycline or co-amoxiclav if this is contraindicated for any reason (54, 73, 74).

It should also be noted that a wide range of estimates of infectious dose required to cause infection in humans highlight not only the pathogenic potential of these organisms but also the difficulties of extrapolating animal model data to human risk assessment. The heterogeneity across models underscores the importance of careful interpretation when evaluating infectious dose data, particularly in guiding laboratory risk assessments and the development of PEP guidelines (75–77). Given this uncertainty, biosafety policies and PEP recommendations must adopt conservative assumptions, ensuring adequate protection of laboratory personnel until more precise human-relevant data become available.

### Disinfection and decontamination

Although PEP offers an individual-level intervention, environmental decontamination is critical to prevent persistence and secondary exposures. Beyond preventing exposures, decontamination is crucial to reducing the persistence of *Burkholderia* in laboratory environments. Table 3 summarizes the range of chemical, gaseous, and physical methodologies evaluated for the inactivation of *B. pseudomallei* and *B. mallei*. Among chemical disinfectants, chlorine-based treatments, pH-adjusted bleach, ethanol, quaternary ammonium compounds, phenolic agents such as Pine-Sol, and commercial products including Virkon, Perasafe, and Umonium^38^ consistently demonstrate high efficacy on non-porous surfaces. However, results were more variable on porous materials, such as wood and carpet (59–62, 64, 78, 79). Formaldehyde and paraformaldehyde are effective in inactivating infected cells and tissues, with longer fixation times required for complete penetration of whole organs (65). The WOAH Terrestrial Manual recommends benzalkonium chloride (1/2,000), sodium hypochlorite (500 ppm available chlorine), iodine, mercuric chloride in alcohol, and potassium permanganate as highly effective for *B. mallei* disinfection, while phenolic disinfectants are less effective (80).

Gas-phase decontamination approaches, including hydrogen peroxide and peracetic acid vapor, are endorsed for laboratory fumigation, while formaldehyde fumigation is now largely of historical interest, having been replaced by safer alternatives. Physical methods remain cornerstone practices: heat inactivation occurs rapidly at 74°C–80°C, with autoclaving providing complete sterilization (13, 33, 66). UV irradiation (64, 67) and natural sunlight (68) can achieve substantial reductions in viability, particularly with prolonged exposure, and gamma irradiation (69) is validated for sterilizing research materials.

Collectively, these findings highlight that a wide array of interventions can reliably inactivate *B. pseudomallei and B. mallei*, provided that concentration, contact time, and material type are carefully considered. Ultimately, the choice of method should strike a balance between efficacy, cost, safety, and local availability, particularly in resource-limited settings where sustainable and accessible approaches are essential.

### Sustainable practices in endemic and/or resource-limited settings

#### Biosafe practices in diagnostic laboratories

Guidance for diagnostic and research activities with infectious materials, including those in the CDC’s BMBL6 and the WHO Laboratory Biosafety Manual (fourth edition), emphasizes the use of biological safety cabinets, PPE, and proper waste management to mitigate exposure risks (1, 33). However, variations in implementation, particularly in resource-constrained settings, reflect discrepancies in laboratory infrastructure, training, and adherence to risk-based assessments. While LAIs involving *B. pseudomallei* and *B. mallei* remain rare, case studies underscore the importance of robust training and containment measures. For example, incidents such as needlestick injuries, centrifuge accidents, and aerosol exposures have demonstrated that lapses in containment protocols can lead to infections despite relatively low LAI case numbers (43, 51). Also, it is clear that washing glass Petri dishes containing *B. pseudomallei* for re-use without prior autoclaving constitutes a very high-risk activity and should be avoided at all costs. Furthermore, the underreporting of LAIs, especially in endemic regions, remains a concern (53, 56).

Distinguishing between guidelines for research laboratories and those for routine clinical diagnostic laboratories requires pragmatic considerations. Handling *B. pseudomallei* and *B. mallei* in diagnostic laboratories requires a careful balance between international biosafety recommendations and the realities of laboratories operating in endemic, resource-limited settings. In such settings, the use of additional, and potentially unnecessary, containment potentially complicates the procedures for confirming the identification of *B. pseudomallei*, delaying diagnosis and treatment. In these diagnostic laboratories, initial inoculation of cultures from potentially infectious specimens should follow BSL-2 practices, with strict use of primary containment devices, such as biological safety cabinets, sealed centrifuge cups, or safety rotors, to minimize aerosol generation (1, 33). Where biosafety cabinets are not consistently available, alternative measures, including containment isolators, splash shields, or acrylic barriers, can be employed, supported by validated SOPs for handling, processing, and disposal. In practice, many diagnostic laboratories in Southeast Asia and northern Australia adopt a risk-based approach, working at BSL-2 with additional safeguards.

In some cases, all blood culture subcultures and suspected *B. pseudomallei* colonies are inspected and manipulated within a biological safety cabinet until identification and susceptibility testing are complete. However, in Australia, diagnostic laboratories, particularly in melioidosis-endemic areas, regularly encounter *B. pseudomallei* in non-sterile site specimens, such as sputum, swabs, and pus. In such settings, *B. pseudomallei* has been safely handled on the open bench in BSL-2 laboratories for many years without any documented *B. pseudomallei* LAIs (55), and given the centralized nature of healthcare in remote regions, the likelihood of under-reporting is low. This reflects a broader practical reality in endemic settings, where diagnostic workflows often require handling culture plates on the bench, and strict containment protocols may not be feasible during routine processing. Centralization of diagnostic testing necessitates the safe and timely transport of clinical specimens and isolates. While international regulations provide guidance on packaging and transport, implementation varies widely, and transport-associated delays may adversely affect diagnosis and patient management in endemic regions. Practical guidance that balances regulatory compliance with diagnostic timeliness is therefore essential, particularly in resource-limited settings.

This pragmatic approach, consistent with the WHO Laboratory Biosafety Manual (fourth edition), emphasizes reliance on good microbiological practices, appropriate PPE, and regular training in emergency response, given that human error remains the leading cause of laboratory incidents (51, 54, 64). Specific attention should be paid to preventing high-risk incidents, such as centrifuge accidents and needlesticks. Innovative measures, such as modular containment systems, retrofitted laboratories with enhanced filtration capabilities, and portable biosafety cabinets, provide additional safeguards when a full BSL-3 infrastructure is not feasible. It will be appreciated that these devices may be prohibitively costly in resource-limited regions. Ultimately, fostering regional collaboration through shared BSL-3 facilities provides a sustainable model to support high-risk activities while enhancing biosafety capacity. Including these practical, locally adapted strategies is essential, as they represent the most realistic and valuable guidance for diagnostic laboratories managing *B. pseudomallei* and *B. mallei* in endemic areas. Importantly, highlighting this gap between international standards and real-world practice underscores the need for policymakers, funders, and health authorities to prioritize investment in sustainable, risk-based biosafety solutions tailored to resource-limited regions.

#### Veterinary and One Health considerations

A One Health approach is essential for understanding and mitigating the risks associated with *B. pseudomallei and B. mallei*, particularly *B. mallei*, which primarily affects horses but can also spread to humans and other animals. In areas where melioidosis is prevalent as a soil-dwelling organism, *B. pseudomallei* causes illness in livestock, pets, and wild animals, including non-human primates, making surveillance and control efforts more challenging (81). Veterinary diagnostic and research laboratories that handle clinical samples, infected animals, or perform necropsies face biosafety challenges similar to those in human health laboratories, often with fewer resources. On occasion, the importation of animals infected with *B. pseudomallei* into non-endemic areas has prompted a vigorous public health response (82). Past outbreaks of glanders and melioidosis show the zoonotic risk and occupational hazards for vets, animal handlers, and lab workers. WOAH standards emphasize biosafety and biosecurity in veterinary settings (3, 80, 81); however, applying these standards in resource-limited countries remains inconsistent. Strengthening veterinary laboratory biosafety, enhancing disease surveillance, and incorporating reporting into human health systems are key parts of a sustainable One Health strategy to manage the risks posed by *B. pseudomallei and B. mallei*.

#### Biosecurity and biocontainment

While sustainable laboratory practices are essential to maintaining biosafety in resource-limited contexts, parallel biosecurity challenges persist, particularly regarding Tier 1 select agent oversight, pathogen repositories, and dual-use concerns. The classification of *B. pseudomallei* and *B. mallei* as Tier 1 Select Agents in the United States reflects both their potential for deliberate misuse and their designation as Category B biothreat pathogens, necessitating stringent biosecurity oversight (9, 83). In the United States, this designation requires rigorous regulatory control over possession, transfer, and use, including detailed documentation of every instance of acquisition, manipulation, storage, and destruction of isolates. Such measures include establishing secure biorepositories, where pathogens and their derivatives must be carefully tracked through robust inventory systems to ensure accountability at every stage of their life cycle (84). It should be noted that *B. pseudomallei* is now recognized as being endemic in the environment of the southeastern United States (85) and, as such, the rationale for stringent biosafety and biosecurity controls should be questioned and reevaluated.

It should be noted that the significant infrastructure and costs associated with high-level containment facilities and compliance reporting can hinder endemic country laboratories from conducting essential diagnostic, epidemiological, and therapeutic research (86). This imbalance risks centralizing control of valuable reference strains and research capacity in high-income countries, while limiting the ability of endemic nations to generate locally relevant knowledge and maintain autonomy over pathogens of national importance. To reconcile security with scientific progress, sustainable, risk-based approaches are needed that safeguard against the misuse of biothreats while enabling the responsible establishment of regional biorepositories and strengthening local capacity for the safe storage, use, and destruction of these agents (84, 87).

While biosecurity frameworks strengthen global security, they also introduce significant challenges for resource-constrained endemic regions (87), where these organisms naturally occur in soil and water, and where local expertise is crucial for research and outbreak response (8, 39). Considering all the evidence, implementing Select Agent or SSBA-level precautions in endemic, low-resource settings would be impractical and unsustainable (56).

#### Dual-use research of concern considerations

Research on *B. mallei* and *B. pseudomallei* offers important benefits, including the development of vaccines, new therapeutics, improved diagnostics, enhanced disease surveillance, and insights into their environmental persistence. However, the same work also carries “dual-use research of concern” (DURC) risks: knowledge of virulence, transmission, or resistance mechanisms could be misapplied to engineer more lethal, transmissible, or treatment-resistant strains, or to evade detection. Given *B. mallei*’s history of weaponization (14, 88) and recognition of both species as Tier 1 select agents, research on these pathogens exemplifies both the promise of scientific progress and the concerns of DURC. Research on *B. pseudomallei* is already constrained by dual-use considerations: one recent review notes that “dual-use concerns in select agent pathogens such as *B. pseudomallei* mean that it is not possible to induce antimicrobial resistance in the laboratory setting, limiting opportunities to define new resistance mechanisms” (89). Additionally, *B. mallei* and *B. pseudomallei* are explicitly recognized under Institutional Oversight of Life Sciences Dual Use Research of Concern policies (90), placing them under special review in many jurisdictions. Despite formal recognition in policy frameworks, there is a paucity of experimental or conceptual literature on the dual-use risks posed by *B. pseudomallei and B. mallei* compared to viral or synthetic biology agents. This gap suggests a need for future research, oversight guidelines, and publication policies tailored to bacterial Tier-1 agents.

## CONCLUSION

This review highlights the complex biosafety and biosecurity challenges posed by *B. pseudomallei* and *B. mallei*. Despite the availability of methods, numerous challenges complicate risk management. These pathogens carry significant risks due to their high virulence, environmental resilience, and potential use as bioterrorism agents. Although advances have been made in understanding the laboratory and clinical management of these bacteria, critical gaps persist in areas such as risk classification and biosafety protocols, especially in low-resource settings where melioidosis is endemic.

We recommend prioritizing (i) harmonizing biosafety and biosecurity standards across jurisdictions to ensure consistent and practical application worldwide; (ii) developing evidence-based PEP guidelines, supported by clinical data, to enhance management of recognized and unrecognized exposures; (iii) designing and disseminating affordable, scalable containment and decontamination solutions tailored to resource-limited, endemic regions, thereby supporting sustainable laboratory capacity; and (iv) strengthening systematic surveillance and transparent reporting of LAIs to improve risk assessment, inform training, and reduce underreporting. Collectively, these actions will not only mitigate laboratory risks from *B. pseudomallei* and *B. mallei* but also help balance global security requirements with equitable research opportunities, empowering endemic regions to address local threats while contributing to international preparedness.

## Data Availability

There is no specific data set related to this study.

## AFTER EPUB

*[This article was published on 11 March 2026 with the incorrect Global Advisory Board member name in the Ed. Note. The note was updated in the current version, posted on 1 May 2026.]*

## EDITOR'S NOTE

**Ed. Note**: The submission of this paper was coordinated by CMR Global Advisory Board member Shruti Malik. The Global Advisory Board, independent of the peer review process, provides strategic direction for the journal in covering topics of both worldwide and regional interest. For more information, see the Editor in Chief's 2024 editorial.
